# ENRICHING EARLY-LIFE ACTIVITIES AND LATER-LIFE COGNITION: THE MODERATING ROLE OF MULTIPLE SOCIAL POSITIONS

**DOI:** 10.1093/geroni/igae098.2098

**Published:** 2024-12-31

**Authors:** Addam Reynolds

**Affiliations:** University of Southern California Leonard Davis School of Gerontology, Los Angeles, California, United States

## Abstract

Mounting evidence suggests that associations between early life factors and later life cognition are not universal across racialized populations. While evidence suggests that enriching early life activities (EELA) are protective for cognitive aging, no study has evaluated if this association is equally salient for older Black adults across the socioeconomic gradient. Using data from the Life History Mail Survey in the Health and Retirement study, we employ a growth curve analysis to estimate the association between EELA and later life cognition among non-Hispanic White and Black older adults. We also test for moderating effects to assess the relative importance of race and childhood socioeconomic status (cSES) in this association. I found evidence of a three-way interaction between, EELA, cSES and race (b = 0.26, p <.05). Among non-Hispanic White older adults, we observed a stronger relationship between EELA and baseline cognition among those with lower levels of cSES. Among Black participants, we observed the opposite effect. Among Black participants with one standard deviation below the mean of cSES, there was no effect of EELA on baseline cognition. This study suggests that associations between EELA and later life cognition are conditional on multiple social positions, rather than being universal across populations. While White individuals with lower cSES have the most to gain from EELA, Black individuals from similar backgrounds do not benefit from EELA. The implication of this finding suggests that EELA may buffer against the negative effects of lower cSES among White, but not Black participants.

